# The Comparison of the Effects of Education Provided by Nurses on the Quality of Life in Patients with Congestive Heart Failure (CHF) in Usual and Home-Visit Cares in Iran

**DOI:** 10.5539/gjhs.v6n3p256

**Published:** 2014-04-11

**Authors:** Hosseinali Mehralian, Shahriar Salehi, Jafar Moghaddasi, Masoud Amiri, Hossein Rafiei

**Affiliations:** 1 Department of Medical Surgery, School of Nursing and Midwifery, Shahrekord University of Medical Sciences, Shahrekord, Iran; 2 Social Health Determinants Research Center, Shahrekord University of Medical Sciences, Shahrekord, Iran

**Keywords:** quality of life, education, nurse, home-visit, congestive heart failure

## Abstract

**Aim:**

Quality of life (QOL) can be considered as a quality indicator of health care systems and nurses can play an important role to improve QOL in patients with congestive heart failure (CHF). The aim of this study was to determine the effects of education provided by nurses on the QOL in patients with CHF in home-visit care compared to usual care.

**Method:**

In a single-blind randomized controlled trial conducted from September 2011 to June 2012, 110 patients with CHF were randomly assigned into two groups. While patients in group I were received usual education at the time of hospital discharge, patients in Group II, in addition to usual education, were received special education regards to their illness by nurses who visited patients in their homes. The 36-item short-form (SF-36) questionnaire was used to evaluate the patient’s QOL at the time of discharge and also six months after hospital discharge.

**Results:**

At the time of hospital discharge, mean score of all 8 sub-score of SF-36 questionnaire was 63.4±7.8 in patients of group II and 61.1±6.4 in patients of group I, respectively (P> 0.05). QOL was decreased in group I and increased in group II compared to the time of hospital discharge. After six months, mean score of QOL was higher in group II than in group I.

**Conclusions:**

QOL of patients with CHF were decreased after hospital discharge. Education provided by nurses in home-visit care could improve the QOL in patients with CHF, based on the findings of this study.

## 1. Introduction

Incidence of heart diseases has increased from 5.8% to 16.8% from 1995 to 2001 in Iran (Iranian Ministry of Health and Medical Education, 2010) which one of the most common forms of heart diseases is congestive heart failure (CHF) (Coelho et al., 2005; Fonarow, 2004; Sadeghi Sherme, Ahmadi, Babatabar, & Haji Amini, 2009; Saccomann et al., 2011; Murray-Thomas, & Cowie, 2005). In fact, about 15 million patients throughout the world and 4.9 million patients in the United States (US) may suffer from CHF (Carelock & Clark, 2001; Sadeghi Sherme et al., 2009). Furthermore, according to the report of Iranian health care agency in 2001, the rate of CHF was 3.3 in every 100 people (Iranian Ministry of Health and Medical Education, 2010). It means that the rate of readmission among patients with CHF is high and may impose the health care systems with many further problems (Hou et al.,2004).

The physical restrictions and advanced symptoms derived from this disease might result in decreasing the QOL in patients with CHF (Sadeghi Sherme et al., 2009). Compared to other chronic diseases such as rheumatoid arthritis and chronic obstructive pulmonary disease, negative effects of CHF on patients’ QOL are substantial. Yousefi, Sabzevari, Mohammadalizade and Haghdoost studied the QOL of patients with CHF in Iran in 2011 and reported that these patients had not appropriate QOL. They have also found that illiterate patients and women had lower QOL. In another study in Iran, Rahnavard, Zolfaghari, Kazem Nejad and Hatami Poor (2007) examined the QOL and its related risk factors in patients with CHF. They found that almost half of patients had lower levels of QOL in physical, psychological, social and economical dimensions. They also reported that the education, job, severity of disease, number of readmission, edema, high level of blood lipids and fatigue during activity could impact on QOL among patients with CHF. Study of Juenger et al. (2002) in Germany showed that New York Heart Association (NYHA) functional class is most dominant predictor of changing the QOL in patients with CHF. They also reported that there was no clear association between QOL and left ventricular ejection fraction (LVEF), duration of disease, and age. In Sweden, Martensson, Stromberg, Dahlstrom, Karlsson and Fridlund (2005) studied on the effects of a nurse-led intervention on QOL and depression in 153 patients with CHF and reported that nurse-led intervention in a primary health care setting had limited effects on the QOL and depression in these patients.

QOL could be considered as a quality indicator of health care systems. One of the main goals of caring of CHF patients is QOL improvement (Yousefi et al.,2011). It is obvious that to achieve this goal, nurses have an important role. However, in Iran the role of nurses in improvement of QOL in CHFs outpatients has not received enough attention. In our study, the main aim was to assess the effects of education provided by nurses in home-visit care compared to regular education at the time of hospital discharge on QOL in patients with CHF.

## 2. Materials and Methods

This single-blind randomized controlled trial conducted from September 2011 to June 2012 in Shahrekord, southwest of Iran. The study has been obtained permission from the Ethics Board of the Shahrekord University of Medical Science. Inclusion criteria were: having CHF diagnosed by cardiologist, age more than 18 years, NYHA class II–IV, ejection fraction less than 45%, ability to reading and writing and accept to participant in study. Patients with history of other diseases who needed to have a surgery during study period and psychological disorder were excluded. Each patient was asked to fill in a written consent form.

One hundred ten patients randomly assigned in two groups. Patients in group I, were received usual education provided by nurses in the time of hospital discharge (one hour before patients discharge, nurses visited patients in their room and answered the questions of the patients and their family). In addition to the usual education in the time of hospital discharge and during home visits, patients in group II were received special education regards to their illness by nurses who visited patients in their homes. Special education according to the checklist of home-visit care in CHF patients was included: information about their disease, usual signs and symptoms and potential complications of their illness, prescribed medications, potential change in their lifestyle, special signs and symptoms which they have to know in order to go to the hospital on time and any other information about the illness which patients may request to be answered. Patients in group II also received one simplified booklet about CHF. Patients’ education in both groups provided by nurses whom had more than five years experience in caring of CHF patients. Home-visits were scheduled two times per month in 1, 3 and 6 months after patients’ discharge from hospital. Patients and their families were encouraged to make contact in the event of problems to their condition.

QOL of patients was measured by the Iranian version of the 36-item short form (SF-36) questionnaire at discharge time and also six months after hospital discharge (Thompson, Roebuck, & Stewart, 2005). The SF-36 is a generic multidimensional instrument consisting of eight multi-item components representing physical functioning (PF; the extent to which health limits physical activities, such as self care, walking and climbing stairs), role of physical functioning (RP; the extent to which physical health interferes with work or other daily activities), bodily pain (BP; the intensity of pain and the effect of pain on normal work, both inside and outside the home), general health perceptions (GH; personal evaluations of current health, health outlook, and resistance to illness), vitality (VT; feeling full of energy rather than tired and worn out), social functioning (SF; the extent to which physical health or emotional problems interfere with normal social activities), role of emotional functioning (RE; the extent to which emotional problems interfere with work or daily activities) and mental health (MH; general mental health including depression, anxiety, behavioral-emotional control, and general positive affect). SF-36 scores were converted to a scale of 0 to 100, in which a higher score is indicating a better QOL. Baseline demographic and clinical characteristics including age, sex, medical history, the establishment of the diagnosis, and prescribed medication were extracted from the patients’ medical records. The data analysis was performed using SPSS (Statistical Package for the Social Sciences) version 17. A P-value of less than 0.05 was considered as statistically significant. Descriptive statistics (expressed as mean and standard deviation (SD), paired T-test and independent T- test for comparing the means of normally distributed independent-samples were used.

## 3. Results

Of 55 patients of group I, 4 patients died and one patient rejected to continue the study (Table 1). In group II, 2 patients died and 4 patients withdrew due to their own request. 62.2% of patents in group I and 54% of patients in group II were men. Mean age of patients in group I and II were 62.7±10 and 61.28±13, respectively. Most of the patients were married: 96% in group I and 100% in group II. According to NYHA criteria, the most prevalent CHF degree was class III in both groups (82% in group I and 67.3% in group II). At the time of hospital discharge, mean score of all 8 sub-score of SF-36 questionnaire was 63.4±7.8 in group II and 61.1±6.4 in group I, respectively (P>0.05). In both groups, largest score was related to sub-scale of GH and smallest score was related to sub-scale of PF. After six months, mean score of QOL was higher in group II than in group I (P<0.05). In addition, after six months, compared to the time of hospital discharge, QOL was improved in patients of group II (P<0.05). However, in patients of group I, QOL scores were declined after six months (P<0.05). Table 1 shows the comparison of the QOL scores before and after intervention.

**Table 1:** Comparison of the QOL scores before and after intervention

QOL Domain	Groups					
	Before intervention		P value	After intervention		P value
	Group I	Group II		Group I	Group I	
Physical functioning	55.08±8.62	52.22±10.87	p > 0.05	49.92±7.24	55.16±12.19	p < 0.05
Role-physical	55.14±9.91	54.32±12.41	p > 0.05	51.32±7.51	55.74±11.65	p < 0.05
Bodily pain	72.80±16.55	69.10±22.47	p > 0.05	64.43±16.38	72.88±17.96	p < 0.05
General health	73.33±17	72.62±21.93	p > 0.05	67.92±18.50	75.28±19.33	p < 0.05
Vitality	57.43±11.67	56.92±13.62	p > 0.05	54.12±9.76	58.58±13.06	p < 0.05
Social functioning	67.82±15.68	64.62±19.71	p > 0.05	59.90±11.51	66.56±18.74	p < 0.05
Role-emotional	58.84±10.33	57.18±12.39	p > 0.05	56.43±8.67	58.34±12.27	p > 0.05
Mental health	62.90±13.76	61.12±16.83	p > 0.05	59.76±12.28	61.78±16.29	p > 0.05

## 4. Discussion

The QOL is defined as perception of people about their life, values, goals, standards, and interests (Yaghoubi et al.,2012). Our study showed that educations provided by nurses in home-visits may improve QOL more than educations only in discharge time of patients with CHF.

Vavouranakis and colleagues (2003) studied the effect of home-based intervention on hospital readmission and QOL in middle-aged patients with severe CHF. They reported that the use of continuous education and intervention in these patients could increase physical function and decrease physical pain and rate of readmission, which is similar to our findings. In another study, in 2010 in Iran, Sadeghi Sharmeh (2009) studied the effects of applying continuous care model (an Iranian care model) on QOL of patients with heart failure. They reported that applying care continuous model in these patients could enhance QOL in physical, emotional and general dimensions. Furthermore, the results of Chinaglia’s (2002) study were also similar to our results. They examined a nurse-based heart failure management program on hospitalization rate, functional status, QOL and medical costs in patients with CHF and reported that nurses can play an important role in management of patients with CHF.

However, Martensson and colleagues (2007) reported that the use of nurse-led intervention have limited effects on QOL in HF patients in a primary health care setting, which is not similar to our results. This difference might be due to the difference in health care systems of Iran and Sweden. Because in Iran, patients with CHF would receive education provided by nurses only at the time of hospital discharge and home-visits are not performing at this time. Thus, Iranian patients probably have less information about their disease at the time of hospital discharge; therefore, they would have lower levels of QOL compared to Swedish patients. In addition, the different method of two studies may result in the observed difference. In Sweden, educations and follow-up were performed using telephone; however, in our study, instead of using phones, home-visits were done by nurses.

Previous studies in Iran have reported that Iranian patients with CHF had lower level of QOL (Rahnavard et al., 2007; Yaghoubi et al., 2012; Yousefi et al.,2011). They showed that many factors such as age, sex, duration of diagnosis, smoking, positive familial heart diseases decrease, educational status, job, treatment method, cardiac ejection fraction, diabetes and high blood pressure could affect on the QOL in this group of patients. Moreover, we found that usual educations for patients with CHF at the time of hospital discharge were insufficient and QOL decreased in patients with CHF as well as findings of Rahnavard et al. (2007) who reported incompleteness of education provided by nurses at the time of hospital discharge in Iranian hospitals. He suggested that nurses/nursing students should receive more training about giving information to the patients and their families about their diseases, especially in patients with CHF. There was also an emphasis on the important role of nurses to increase the QOL of patients with CHF. In addition, Yousefi and colleagues (2011) also reported that nurses have to pay more attention to QOL in patients with CHF. They suggested that to increase QOL of patients, their follow-up and home-visits after discharge should be considered as an important aspect of nursing care in CHF patients.

## 5. Conclusion

The QOL of patients with CHF may decrease after hospital discharge. Therefore, nurses have an important role in caring of this group of patients. Educations provided by nurses in home-visits could improve the QOL in patients with CHF. Nurses should pay more attention to all aspects of patients’ QOL, especially physical functioning due to its nature to have more decrease after hospital discharge than other dimensions of QOL. Finally, Iranian health care systems are needed to change their programs in term of patients follow-up and home-visits after hospital discharge.
